# In Vitro Antimicrobial Susceptibility Profiles of Gram-Positive Anaerobic Cocci Responsible for Human Invasive Infections

**DOI:** 10.3390/microorganisms9081665

**Published:** 2021-08-04

**Authors:** François Guérin, Loren Dejoies, Nicolas Degand, Hélène Guet-Revillet, Frédéric Janvier, Stéphane Corvec, Olivier Barraud, Thomas Guillard, Violaine Walewski, Emmanuelle Gallois, Vincent Cattoir, on behalf of the GMC Study Group

**Affiliations:** 1 Service de Bactériologie-Hygiène Hospitalière, CHU de Rennes, F-35033 Rennes, France; francois.guerin@chu-rennes.fr (F.G.); loren.dejoies@chu-rennes.fr (L.D.); 2 Laboratoire de Bactériologie, CHU de Nice, F-06202 Nice, France; nicolasdegand@free.fr; 3 Laboratoire de Bactériologie-Hygiène, Hôpital Purpan, F-31059 Toulouse, France; guet-revillet.h@chu-toulouse.fr; 4 Service de Microbiologie et Hygiène Hospitalière, Hôpital d’Instruction des Armées Saint-Anne, F-83800 Toulon, France; janvierfrede@gmail.com; 5 Service de Bactériologie et des Contrôles Microbiologiques, CHU de Nantes, F-44093 Nantes, France; stephane.corvec@chu-nantes.fr; 6 Laboratoire de Bactériologie-Virologie-Hygiène, CHU Dupuytren, F-87042 Limoges, France; olivier.barraud@unilim.fr; 7 Laboratoire de Bactériologie-Virologie-Hygiène Hospitalière, Hôpital Robert Debré-CHU de Reims, F-51090 Reims, France; tguillard@chu-reims.fr; 8 Service de Microbiologie, Hôpitaux Universitaires de Paris Seine Denis (HUPSSD), Site Avicenne, AP-HP, F-93000 Bobigny, France; violaine.walewski@aphp.fr; 9 Service de Microbiologie, Gustave Roussy, F-94805 Villejuif, France; emmanuelle.gallois@gmail.com

**Keywords:** GPAC, antimicrobial resistance, *Finegoldia magna*, *Parvimonas micra*, *Peptostreptococcus anaerobius*, *Peptoniphilus*, *Anaerococcus*

## Abstract

The aim of this multicentre study was to determine the in vitro susceptibility to anti-anaerobic antibiotics of Gram-positive anaerobic cocci (GPAC) isolates responsible for invasive infections in humans. A total of 133 GPAC isolates were collected in nine French hospitals from 2016 to 2020. All strains were identified to the species level (MALDI-TOF mass spectrometry, 16S rRNA sequencing). Minimum inhibitory concentrations (MICs) of amoxicillin, piperacillin, cefotaxime, imipenem, clindamycin, vancomycin, linezolid, moxifloxacin, rifampicin, and metronidazole were determined by the reference agar dilution method. Main *erm*-like genes were detected by PCR. The 133 GPAC isolates were identified as follows: 10 *Anaerococcus* spp., 49 *Finegoldia magna*, 33 *Parvimonas micra*, 30 *Peptoniphilus* spp., and 11 *Peptostreptococcus anaerobius*. All isolates were susceptible to imipenem, vancomycin (except 3 *P. micra*), linezolid and metronidazole. All isolates were susceptible to amoxicillin and piperacillin, except for *P. anaerobius* (54% and 45% susceptibility only, respectively). MICs of cefotaxime widely varied while activity of rifampicin, and moxifloxacin was also variable. Concerning clindamycin, 31 were categorized as resistant (22 *erm*(A) subclass *erm*(TR), 7 *erm*(B), 1 both genes and 1 negative for tested *erm* genes) with MICs from 8 to >32 mg/L. Although GPACs are usually susceptible to drugs commonly used for the treatment of anaerobic infections, antimicrobial susceptibility should be evaluated in vitro.

## 1. Introduction

Gram-positive anaerobic cocci (GPAC), which are a major part of the normal human microbiota of the oropharynx, respiratory tract, skin, and urogenital and gastrointestinal tract (GIT), are frequently recovered from human specimens, accounting for 25–30% of all anaerobic clinical isolates [1,2]. They are also opportunistic pathogens responsible for many human infections (often polymicrobial) especially in the elderly population and immunocompromised patients, including pleuropulmonary, intraabdominal, pelvic, skin, and soft-tissue and bone and joint infections (BJIs) as well as brain abscess and bacteremia [1,2,3,4].

GPAC has long been an heterogenous group of organisms and then has undergone considerable taxonomic changes with the creation of new genera formed from species previously belonging to the genus *Peptostreptococcus*, such as *Finegoldia*, *Parvimonas*, *Anaerococcus,* and *Peptoniphilus* while the only remaining representative of this genus is *P. anaerobius* [5]. Other changes within the GPAC group include the addition of new species in these new genera. The most commonly species found in clinical material are *Finegoldia magna*, *Parvimonas micra*, *Peptoniphilus harei*, and *Peptostreptococcus anaerobius* [2,6,7].

Only few studies have focused on in vitro antimicrobial susceptibility of GPAC isolates, many of them using unreliable conventional identification methods based on biochemical tests. In addition, the published data are often based on GPAC in general (formerly *Peptostreptococcus* spp.) even though there are significant differences in antibiotic susceptibility between GPAC species [2]. Indeed, it is now accepted to identify to the species level GPAC isolates in clinical specimens for susceptibility testing to adapt the correct antibacterial therapy, which is now possible with the development and application of molecular methods and MALDI-TOF mass spectrometry [2,6,8,9]. Even if it is widely accepted that these microorganisms remain consistently susceptible to antimicrobial agents generally used for the treatment of such anaerobic infections, antimicrobial resistance among anaerobes has increased in recent years worldwide and clinical failures have been reported in patients receiving inappropriate treatments [10,11].

The aim of the study was to investigate the in vitro antimicrobial susceptibility of a large collection of GPAC clinical isolates responsible for human invasive infections to 10 antimicrobial agents as well as to decipher the molecular basis of clindamycin acquired resistance.

## 2. Materials and Methods

### 2.1. Bacterial Isolates and Identification

A total of 133 non-redundant clinical isolates of GPAC collected from patients suffering from invasive infections in nine French hospitals between 2016 and 2020, including 109 (82%) from 2019, were studied. These isolates were recovered from patients with bacteremia (*n* = 37; 28%), BJIs (*n* = 51; 38%), and deep-seated soft-tissue infections (*n* = 45; 34%). For microbiological investigation, strains were grown on 5% horse blood agar plates (bioMérieux, Marcy-l’Etoile, France) incubated for at least 48 h in an anaerobic chamber at 35 °C. Phenotypic identification at the species level was performed using the MALDI-TOF mass spectrometry technology (Microflex; Bruker Daltonics, Wissembourg, France) in accordance with the manufacturer’s instructions, and if necessary, by sequencing of the 16S rRNA gene, as previously described [12].

### 2.2. Antimicrobial Susceptibility Testing (AST)

MICs were determined using the reference agar dilution method according to 2018 CLSI guidelines using Brucella agar supplemented with 5% laked sheep blood, 5 mg/L hemin, and 1 mg/L vitamin K [13]. Plates were inoculated using a Steers replicator device (delivering a final inoculum of ca. 10^5^ CFU per spot) and incubated anaerobically for 48 h at 35 °C. The 10 following antibiotics were tested: amoxicillin, piperacillin, cefotaxime, imipenem, clindamycin, vancomycin, linezolid, moxifloxacin, rifampicin, and metronidazole. MICs were interpreted according to 2020 CA-SFM/EUCAST clinical breakpoints recommended for anaerobes except for cefotaxime for which the PK-PD (non-species related) breakpoint was used (https://www.sfm-microbiologie.org/, accessed on 25 March 2021). *Bacteroides thetaiotaomicron* ATCC 29741 and *Clostridioides difficile* ATCC 700057 were used as quality control strains. The production of β-lactamase was assessed using the nitrocefin disk (Cefinase, BD BBL) as recommended by the manufacturer.

### 2.3. Detection of Resistance Genes

The bacterial genomic DNA of macrolide-resistant isolates was extracted using the QIAamp DNA Mini Kit (Qiagen, Courtaboeuf, France). Detection of *erm*(A) [including subclass *erm*(TR)], *erm*(B), *erm*(C), *erm*(F), *erm*(T) and *erm*(X) genes was performed by PCR as previously described [14]. All PCR-amplified products were sequenced in both directions by the Sanger method using the same primers.

## 3. Results

**Figure 1:**
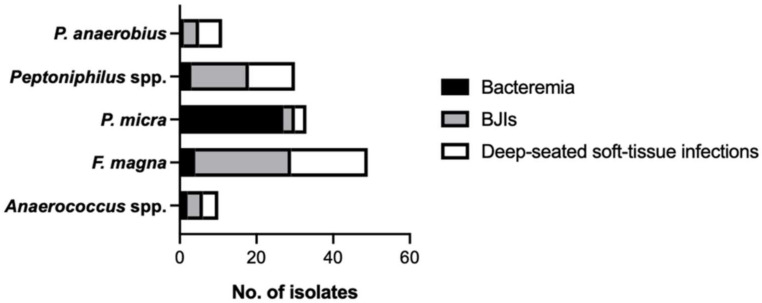
Distribution and origin of the different Gram-positive anaerobic cocci (GPAC) clinical isolates.

Of the 133 GPAC clinical isolates, the most frequently isolated species was *F. magna* (*n* = 49; 36.8%) followed by *P. micra* (*n* = 33; 24.8%), *Peptoniphilus* spp. (*n* = 30; 22.6%), *P. anaerobius* (n = 11; 8.3%), and *Anaerococcus* spp. (*n* = 10; 7.5%) (Figure 1). Six different species were identified among *Peptoniphilus* spp. (19 *P. harei* (63.3%), 4 *P. indolicus*, 3 *P. grossensis*, 2 *P. lacrimalis*, 1 *P. gorbachii*, 1 *P. assacharolyticus*) while three were in *Anaerococcus* spp. (8 *A. vaginalis* (80%), 1 *A. octavius*, 1 *A. nagyae*). Note that *P. micra* isolates were mostly (82%) recovered from bacteremia while a majority (≥50%) of isolates of *F. magna* and *Peptoniphilus* spp. were collected from BJIs (Figure 1).

Regardless of the bacterial species, all 133 isolates tested were categorized as susceptible to imipenem, linezolid, and metronidazole (Table 1). Surprisingly, three strains of *P. micra* (3/33; 9%) were categorized as resistant to vancomycin (MIC = 4 mg/L) whereas all other 130 isolates were susceptible (Table 1). Except 5/11 (45%) resistant strains of *P. anaerobius* (MIC = 8–16 mg/L), all other 128 strains were susceptible to amoxicillin with MICs ranging from ≤0.03 to 2 mg/L (Table 1). None isolate exhibited a β-lactamase activity. Only seven isolates were not susceptible to piperacillin (1 *F. magna*, MIC = 16 mg/L; 6 *P. anaerobius*, MIC = 16–32 mg/L) while 57 isolates (2/10 *Anaerococcus* spp., 44/49 (90%) *F. magna*, 1/33 *P. micra*, 4/30 *Peptoniphilus* spp., 6/11 (55%) *P. anaerobius*) exhibited MICs higher than 2 mg/L (Table 1). It is noteworthy that all the 6 cefotaxime-resistant (MIC = 8 mg/L) *P. anaerobius* isolates were also highly-resistant to amoxicillin (MIC = 8–16 mg/L) and piperacillin (MIC = 16–32 mg/L) whereas other cefotaxime-resistant isolates were susceptible to both penicillins (except 1 *F. magna*). Most of strains (80–100%) were susceptible to rifampicin with only 10 non-susceptible strains (2 *Anaerococcus* spp. 6 *F. magna*, 1 *P. micra*, 1 *Peptoniphilus* spp.) (Table 1). Moxifloxacin seemed to have a limited activity against *F. magna* (21/49 (43%) with MIC ≥ 4 mg/L) while other species were mostly (80–91%) categorized as susceptible (Table 1).

Concerning clindamycin, 31 were categorized as resistant (MIC = 8–>32 mg/L) including 2 *Anaerococcus* spp. (2 *A. vaginalis*), 12 *F. magna*, 2 *P. micra*, 14 *Peptoniphilus* spp. (6 *P. harei*, 3 *P. indolicus*, 2 *P. lacrimalis*, 2 *P. grossensis* and 1 *P. gorbachii*), and 1 *P. anaerobius* (Table 1 and Table 2). Of them, 22 and 7 possessed acquired *erm*(A) subclass *erm*(TR) and *erm*(B) genes, respectively, while one strain was positive for both *erm*(A) subclass *erm*(TR) and *erm*(B) genes and no *erm*-like gene was found for one strain (Table 2).

## 4. Discussion

In a study using 16S rRNA-targeted probes and/or sequencing of the 16S rRNA gene for accurate identification, Wildeboer-Veloo et al. showed that the most frequently encountered GPAC in human specimens among 188 clinical isolates were *F. magna* (29%), *P. micra* (22%) and *P. harei* (18%) followed by *P. ivorii* (6%), *A. vaginalis* (5%), *A. lactolyticus* (5%), and *P. anaerobius/stomatis* (5%) [6]. A European surveillance study conducted on 299 GPAC reported that the majority of clinical isolates were identified as *F. magna* (37%), *P. micra* (18%), *P. harei* (15%), *A. vaginalis* (7%), and *P. anaerobius* (7%) [15]. Here, we report a similar relative distribution with a proportion of 37% for *F. magna* followed by *P. micra* (25%), *P. harei* (14%), *P. anaerobius* (8%), and *A. vaginalis* (6%) (Figure 1), which confirms the clinical importance of these Top5 species.

As observed here, *F. magna* is the most common species of GPAC recovered from human clinical specimens, accounting for 5–12% of all anaerobic isolates and 20–38% of all GPAC isolates [1]. It is also likely the most pathogenic organism and the species most frequently isolated in pure culture [11]. Typical infections due to this species are soft-tissue abscesses, wound infections (incl. diabetic ulcers, pressure ulcers), and BJIs [5].

*P. micra* is part of the normal commensal microbiota of the GIT and the gingival crevice and it is mainly recognized as an oral pathogen especially isolated from polymicrobial infections such as periodontitis while it has been implicated in infections in other parts of the body [2,5]. In our study, it was mainly (82%) isolated from blood that is concordant with its frequent implication in GPAC bacteremia in a recent Swedish study, representing 42% (96/226) of GPAC episodes [7]. The authors also showed that GPAC bacteremia is much more common than previously reported and is a condition with significant 30-day mortality (11%) mainly in elderly patients with comorbidities [7].

As described here, *Peptoniphilus* spp. (mainly *P. harei*) are found in chronic wound samples (e.g., ulcer samples, diabetic wounds) and in osteoarticular samples [2]. With *A. lactolyticus*, *A. vaginalis* has been identified among the predominant species in grouped samples of diabetic foot ulcers and pressure ulcers [2]. *P. anaerobius* is recognized as part of the GIT and is one of the most common GPAC associated with infections of the abdominal cavity and female genitourinary tract [1,2].

GPAC are usually susceptible to antibiotics used to treat anaerobic infections but increasing resistance trends have been reported and major differences between species of GPAC have been observed [1,15,16,17,18,19,20,21,22,23,24,25,26,27]. In addition, most AST reports until recently presented data for GPAC as a group rather than for individual species [1,15,28,29,30,31,32].

Most evidence suggests that *F. magna*, *P. micra* and *P. harei* are almost always susceptible to penicillins [16,17,19,20,21,22,23,24,25,26,27,28,29,31,33]. By contrast, strains of *P. anaerobius* usually present higher MIC values and some of them are highly resistant with MICs of amoxicillin up to 16–64 mg/L [16,17,19,26,27,33]. In our study, MICs of amoxicillin and piperacillin were also up to 64- and 256-fold higher for *P. anaerobius* than those for other species, respectively (Table 1). Penicillin resistance seemed to be due not to β-lactamase production but rather to alterations in penicillin-binding proteins (PBPs) as previously reported [34]. The activity of cefotaxime appeared to be moderate with MIC_50_ from 1 to 16 mg/L, especially against *F. magna* and *P. anaerobius* (only 10% and 45% of susceptible strains, respectively) whereas GPAC were uniformly susceptible to imipenem (MIC_50_ ≤ 0.12 mg/L) as previously reported [15,16,17,19,20,25,26,28,29,31,35].

Resistance to macrolides and lincosamides was described in *Peptostreptococcus* spp. three decades ago [36]. As observed here, clindamycin resistance rates among GPAC vary widely and this resistance is rising, especially in *F. magna* (3–51%) and *Peptoniphilus* spp. (1–36%) for which MIC_90_ range from 2 to >256 mg/L [11,16,17,19,20,22,23,25,26,37]. In a recent global program (T.E.S.T. 2010–2016) evaluating the in vitro activity of tigecycline and comparators against a large European collection of anaerobes, susceptibility to clindamycin was 77%, 96%, 87%, 80%, and 95% against *F. magna* (*n* = 654), *P. micra* (*n* = 456), *P. asaccharolyticus* (*n* = 78), *P. harei* (*n* = 209), and *P. anaerobius* (*n* = 256), respectively [38].

Although levels of clindamycin resistance can be as high as 50%, little is known about the genetic basis of resistance to macrolides-lincosamides-streptogramins (MLS) in GPAC. Therefore, only two studies have investigated the molecular basis of MLS basis in these organisms. The first study reported an incidence of 80% macrolide resistance among 21 clinical isolates of *Peptostreptococcus* spp. due to *erm*(A) subclass *erm*(TR), suggesting that these anaerobic members of the normal oropharyngeal microbiota may be an important reservoir of this gene for transfer to pathogens such as *Streptococcus pyogenes* [39]. In the second study, we detected 25 *F. magna* isolates (out of 69; i.e., 36%) that exhibited high-level MICs of erythromycin (>256 mg/L) harboring either *erm*(A) subclass *erm*(TR) or *erm*(B) [40]. Altogether, our findings confirm that *erm*(A) subclass *erm*(TR) is the predominant MLS resistance gene among GPAC and that *erm*(B) could also be detected whereas other *erm*-like genes have never been identified up to now. It is noteworthy that we did not perform the detection of *erm* genes among clindamycin-susceptible isolates, which constitutes a limit of our study since *erm* genes can be harbored by strains categorized as susceptible.

Almost all GPAC isolates are susceptible to metronidazole (as in our study) even if some resistant strains have been described [15,16,17,19,20,23,25,26,28,29,30,31,32,38,41]. Indeed, reduced susceptibility to metronidazole has so far only been rarely detected in surveillance studies among some *P. micra*, *F. magna,* and *Peptostreptococcus* spp. isolates [42]. Several molecular mechanisms have been associated with metronidazole resistance, mainly in *Bacteroides fragilis* [42]. It is mainly related to drug inactivation by nitroimidazole reductase encoded by *nim* genes [42]. To date, 11 *nim* genes (*nimA* to *nimK*) sharing between 54 and 90% amino acid identities have been described [40]. Of them, only *nimB* genes have been identified in the chromosome of some GPAC isolates (*P. anaerobius*, *A. prevotii*, *P. micra*) [42]. Note that the *nimB* gene was detected in 34% (21/61) of GPAC isolates with MIC of metronidazole ≥0.5 mg/L, including only two highly resistant *F. magna* isolates (MIC > 128 mg/L) while the other 19 strains remained susceptible [43]. Since metronidazole is often the drug used for empirical treatment of anaerobic infections and some resistant GPAC clinical isolates have been described, microbiologists should verify the activity of this antibiotic if necessary.

In the literature, no acquired high-level resistance to vancomycin (MIC_50_ = 0.06–1 mg/L; MIC_90_ = 0.25–1 mg/L) or to linezolid (MIC_50_ = 0.5–4 mg/L; MIC_90_ = 0.5–4 mg/L) has been reported so far [15,18,21,23,24,26,27,30,31,35,41]. In our study, three strains of *P. micra* exhibited an MIC of 4 mg/L and were categorized as resistant but they are probably part of the wild-type population, as previously described [31]. All strains were susceptible to linezolid regardless the species (MIC_50_ and MIC_90_ = 0.5–2 mg/L).

In our study, susceptibility to moxifloxacin greatly varied depending on the species (9–43%), which is concordant with observations reported in the literature (0–48%) [20,23,25,26,30,31,32,37]. In agreement with these studies, we also observed that *F. magna* was more resistant than other species. Among newer fluoroquinolones, it was demonstrated that delafloxacin was more active than levofloxacin against GPAC (*n* = 20), with MIC_50_ and MIC_90_ values of 0.003 and 0.38 mg/L versus 2 and >32 mg/L, respectively [44]. In this later study, none of the GPAC isolates tested presented a MIC of delafloxacin equal or higher than 4 mg/L [44].

## 5. Conclusions

In our study, a remarkable difference in antibiotic susceptibility among the five most clinically important GPAC species (i.e., *F. magna*, *P. micra*, *P. harei*, *A. vaginalis*, and *P. anaerobius*) was found. These differences underline the importance to identify to the species level clinical isolates responsible for human infections. Compared to the processing time for aerobic bacteria, the cultivation and identification steps for anaerobic isolates usually take much longer. Clinical laboratories should provide any clinically relevant information in a precise and timely manner to the clinicians since the lack of detection of an infection-associated anaerobic isolate can often lead to inappropriate therapeutic choices and clinical failure [45]. This is particularly true in this period of emergence of antimicrobial resistance among clinically relevant anaerobes. However, in vitro susceptibility testing is not routinely performed because it is time consuming and has some technical issues. For individual patient management, it is important to remember that AST for anaerobes should be performed when: (1) the selection of an active agent is critical for disease management, (2) long-term therapy is being considered, (3) anaerobes are recovered from sterile body sites, (4) the infection persists despite adequate therapy with an appropriate therapeutic regimen, and/or (5) in cases of single recovered anaerobic pathogen from culture [10]. Besides the aforementioned points, susceptibility testing should also be performed whenever it is possible, especially for epidemiological surveillance purposes and to guide the choice of empirical antibiotic therapy [45]. This surveillance (locally, nationally, and internationally) needs to be continuously conducted, since recommendations on first-line agents of therapy are usually based on these data.

## Figures and Tables

**Table 1:** Antimicrobial susceptibility profiles of the 133 GPAC human invasive isolates.

Antibiotic (Clinical BP) ^a^	MIC (mg/L)	*Anaerococcus* spp. ^b^ (*n* = 10)	*F. magna* (*n* = 49)	*P. micra* (*n* = 33)	*Peptoniphilus* spp. ^c^ (*n* = 30)	*P. anaerobius* (*n* = 11)
Amoxicillin (≤4/>8)	MIC_50_	≤0.03	0.25	0.06	0.12	2
	MIC_90_	0.25	0.5	0.5	0.5	16
	Range	≤0.03–0.25	≤0.03–2	≤0.03–2	≤0.03–0.5	0.25–16
	Susceptibility (%)	100	100	100	100	55
Piperacillin (≤8/>16)	MIC_50_	0.12	0.25	0.25	0.06	16
	MIC_90_	2	1	2	2	32
	Range	≤0.03–4	≤0.03–16	≤0.03–4	≤0.03–4	0.25–32
	Susceptibility (%)	100	98	100	100	45
Cefotaxime (≤1/>2)	MIC_50_	0.5	8	0.12	0.12	8
	MIC_90_	16	16	1	2	8
	Range	≤0.03–>16	0.12–>16	≤0.03–8	≤0.03–4	0.25–8
	Susceptibility (%)	80	10	97	87	45
Imipenem (≤2/>4)	MIC_50_	≤0.03	0.06	≤0.03	≤0.03	1
	MIC_90_	0.12	0.25	0.12	0.06	2
	Range	≤0.03–0.12	≤0.03–1	≤0.03–0.25	≤0.03–0.12	≤0.03–2
	Susceptibility (%)	100	100	100	100	100
Clindamycin (≤4/>4)	MIC_50_	0.06	1	0.12	1	0.5
	MIC_90_	16	32	1	>32	1
	Range	≤0.03–>32	≤0.03–>32	≤0.03–>32	≤0.03–>32	≤0.03–>32
	Susceptibility (%)	80	76	94	53	91
Vancomycin (≤2/>2)	MIC_50_	2	2	2	0.5	2
	MIC_90_	2	2	2	2	2
	Range	0.5–2	1–2	0.5–4	0.5–2	1–2
	Susceptibility (%)	100	100	91	100	100
Linezolid (≤2/>4)	MIC_50_	1	2	1	1	0.5
	MIC_90_	1	2	1	2	0.5
	Range	0.5–2	0.25–4	0.5–2	0.25–2	0.5–2
	Susceptibility (%)	100	100	100	100	100
Moxifloxacin (≤1/>2)	MIC_50_	1	0.5	0.25	0.25	0.12
	MIC_90_	2	8	0.5	4	8
	Range	0.25–8	≤0.03–>16	0.03–8	0.06–>16	0.06–8
	Susceptibility (%)	80	57	91	87	82
Rifampicin (≤4/>16)	MIC_50_	≤0.03	0.5	≤0.03	≤0.03	≤0.03
	MIC_90_	>32	>32	≤0.03	1	≤0.03
	Range	≤0.03–32	≤0.03–>32	≤0.03–8	≤0.03–>32	≤0.03–0.5
	Susceptibility (%)	80	88	97	97	100
Metronidazole (≤4/>4)	MIC_50_	1	0.5	0.25	1	0.5
	MIC_90_	2	1	0.5	2	1
	Range	0.25–2	0.25–4	≤0.12–1	0.12–4	0.25–1
	Susceptibility (%)	100	100	100	100	100

^a^ 2020 CA-SFM/EUCAST clinical breakpoints (in mg/L) recommended for anaerobes except for cefotaxime for which PK-PD (non-species related) breakpoint was used. ^b^ 8 *A. vaginalis*, 1 *A. octavius*, 1 *A. nagyae*. ^c^ 19 *P. harei*, 4 *P. indolicus*, 3 *P. grossensis*, 2 *P. lacrimalis*, 1 *P. gorbachii*, 1 *P. assacharolyticus*. MIC: Minimum inhibitory concentration.

**Table 2:** Acquired genes of macrolides-lincosamides-streptogramins (MLS) resistance among the 31 clindamycin resistant GPAC clinical isolates.

Genes	*Anaerococcus* spp. (*n* = 2 ^a^/10)	*F. magna* (*n* = 12/49)	*P. micra* (*n* = 2/33)	*Peptoniphilus* spp. ^b^ (*n* = 14/30)	*P. anaerobius* (*n* = 1/11)
*erm*(A)	−	−	−	−	−
*erm*(A) subclass *erm*(TR)	2	8	−	12	−
*erm*(B)	−	3	2	1	1
*erm*(B) + *erm*(TR)	−	−	−	1	−
*erm*(C)	−	−	−	−	−
*erm*(T)	−	−	−	−	−
*erm*(X)	−	−	−	−	−

^a^ 2/8 *A. vaginalis.* ^b^ 6/19 *P. harei*, 3/4 *P. indolicus*, 2/3 *P. grossensis*, 2/2 *P. lacrimalis*, 1/1 *P. gorbachii.*

## Data Availability

All data are available upon request to the corresponding author.
